# Temperature Prediction of Heating Furnace Based on Deep Transfer Learning

**DOI:** 10.3390/s20174676

**Published:** 2020-08-19

**Authors:** Naiju Zhai, Xiaofeng Zhou

**Affiliations:** 1 Key Laboratory of Networked Control Systems, Chinese Academy of Sciences, Shenyang 110016, China; zhainaiju@sia.cn; 2 Shenyang Institute of Automation, Chinese Academy of Sciences, Shenyang 110016, China; 3 Institutes for Robotics and Intelligent Manufacturing, Chinese Academy of Sciences, Shenyang 110169, China; 4 University of Chinese Academy of Sciences, Beijing 100049, China

**Keywords:** deep learning, temporal convolution network, transfer learning, generative adversarial networks, furnace temperature prediction, multiple heating zones

## Abstract

Heating temperature is very important in the process of billet production, and it directly affects the quality of billet. However, there is no direct method to measure billet temperature, so we need to accurately predict the temperature of each heating zone in the furnace in order to approximate the billet temperature. Due to the complexity of the heating process, it is difficult to accurately predict the temperature of each heating zone and each heating zone sensor datum to establish a model, which will increase the cost of calculation. To solve these two problems, a two-layer transfer learning framework based on a temporal convolution network (TL-TCN) is proposed for the first time, which transfers the knowledge learned from the source heating zone to the target heating zone. In the first layer, the TCN model is built for the source domain data, and the self-transfer learning method is used to optimize the TCN model to obtain the basic model, which improves the prediction accuracy of the source domain. In the second layer, we propose two frameworks: one is to generate the target model directly by using fine-tuning, and the other is to generate the target model by using generative adversarial networks (GAN) for domain adaption. Case studies demonstrated that the proposed TL-TCN framework achieves state-of-the-art prediction results on each dataset, and the prediction errors are significantly reduced. Consistent results applied to each dataset indicate that this framework is the most advanced method to solve the above problem under the condition of limited samples.

## 1. Introduction

As one of the most important pieces of combustion equipment in the metallurgical production process, the heating furnace is also the most important piece of energy consumption equipment in the steel rolling production line. The optimization of the heating furnace is of great significance to iron and steel metallurgical enterprises. The main function of the heating furnace is to heat the billet, make it reach the predetermined temperature, and then roll it [1]. The heating temperature of billet determines the quality of billet. However, the billet temperature cannot be directly measured. Therefore, we take the temperature of the heating furnace collected by the thermocouple sensor as the billet heating temperature. It is difficult to predict the furnace temperature accurately. It is mainly manifested in the following aspects:

- Parameter complexity. There are many kinds of parameters in the production process of a heating furnace, including the structural parameters of the heating furnace (heat exchanger model, thermocouple model), control parameters (furnace temperature of heating section, fan setting opening, gas valve opening), thermal parameters (gas flow, oxygen content, nitrogen flow, steam flow), etc., and the parameters have strong coupling and mutual influence.
- Temperature hysteresis. The heating process is a nonlinear, time-varying, and lagging process. After the implementation of control, the effect will be delayed for a period of time. If the corresponding countermeasures are made after the alarm is triggered, it will cause the loss of production equipment and increase energy consumption. It is necessary to establish a time series prediction model to predict the temperature change trend in advance, so as to adjust the control strategy in time.
- Multi objective prediction. The heating process of billet in the furnace will pass through multiple heating zones. Since different heating zones have independent adjustable control variables, different heating zones correspond to different prediction tasks. At the same time, there is parameter sharing between different heating zones, so the question of how to achieve efficient and accurate prediction of multiple zones is a thorny problem.

In summary, the accurate prediction of furnace temperature is the core and foundation of furnace optimization. The main purpose of forecasting the furnace temperature is to establish a billet heating tracking model to provide the judgment basis for manual operation. If the furnace temperature can be predicted and controlled correctly, the operator can maintain normal fuel distribution. Thus, the operation cost can be minimized, the efficiency of the heating furnace can be optimized, and the service life of the heating furnace can be improved. In addition, another purpose of our study is to present a general algorithm for sensor data analysis of industrial systems with different task requirements.

As mentioned above, a heating furnace is a typical complex industrial control object. The heating process of billet has the characteristics of large lag and large inertia, being multivariable, time-varying, and nonlinear [2]. In addition, it is difficult to accurately predict the temperature distribution in the furnace due to the difficulty in measuring the temperature in the furnace and many external interference factors. Some scholars have tried to solve these problems: the autoregressive integrated moving average model(ARIMA) [3] based statistical learning method and machine learning methods such as support vector regression(SVR) [4] and tree model [5] have been applied to temperature prediction. C. Gao et al. combine fuzzy algorithm and support vector machine to build a fuzzy least square support vector machine to predict temperature [6]. However, the statistical learning method is not suitable for fitting nonlinear and non-stationary data; machine learning methods such as SVR cannot obtain the correlation of data in time series. In contrast, artificial neural network(ANN) has the advantages of being nonlinear and self-learning, which makes the modeling of complex systems simple [7]. Some scholars choose to establish time series models based on a BP neural network for temperature prediction [8]. Chen et al.’s improved extreme learning machine(ELM) [9] method solves the problems of ANN, such as overfitting and slow learning; however, due to the time lag of the heating process, it cannot predict the billet exit temperature [10]. For the defects of the above models applied to the prediction, the deep learning method provides an effective solution.

Multiple hidden layers in the deep learning structure can automatically extract the relevant features and timing information of multiple variables in the heating process, giving the structure a powerful feature learning ability [11]. At present, deep learning technology has been applied to the prediction of furnace temperature. The most widely used methods include the recurrent neural network (RNN) [12] and its variant short-term memory network (LSTM) [13]. However, RNN is prone to the problem of gradient disappearance. LSTM solves the problem of gradient disappearance to a certain extent through the gate control unit, but the training of LSTM needs a lot of data and takes a lot of time. On the other hand, a deep convolution neural network (CNN) has been successfully applied in target classification [14], natural language processing [15], and other applications. CNN is being used to process sequence data by more and more scholars, and the hybrid CNN-LSTM model has achieved magnificent performance in sequence processing [16]. Although RNN and its variants show good performance in sequence processing, as shown in [17] and [18], there are still some problems which need to be solved, such as the fact that it is hard to introspect, difficult to train correctly, and not easy to build an appropriate network architecture for better results. These problems affect the performance of RNN and its variants when applied to furnace temperature prediction.

Recently published research shows that temporal convolutional network (TCN) models perform significantly better than recurrent network structures in sequence modeling problems, including speech analysis and synthesis tasks [19]. Compared to RNN and LSTM, TCN combines casual convolution and dilated convolution [20], and it can capture input sequences of arbitrary length without leaking information. By introducing a residual network mechanism [21], TCN can train deep networks effectively to keep memories longer than LSTM. TCN reduces the training cost through layered sharing of convolution kernels. In terms of structure, TCN does not have the complicated gating structure of LSTM, and the overall framework is simpler. Considering the diversity of furnace variables, we believe that convolutional networks can extract more useful features than RNN and its variants. Due to the lag of furnace temperature prediction and the continuity of the heating process, this study belongs to the category of time series modeling. Based on the above description, we redesigned a TCN structure as the source model to predict the furnace temperature.

In actual production, however, the billet passes through multiple heating zones in the furnace. The furnace temperature of each heating zone is affected by multiple control variables, so the data collected from the thermoelectric coupling sensor will be unstable and nonlinear. Many industrial sensor data have the above characteristics. Since the neural network has no extrapolation, the existing neural network cannot accurately predict such industrial sensor data. In Section 3, we will take the furnace as an example to introduce the data of this type of industrial sensor in detail. Based on the above analysis, it is difficult to accurately predict the temperature of most heating zones in the heating furnace system. In addition, training different models in different heating zones will increase the calculation cost. In view of the above two difficulties, and combined with the characteristics of the similarity of each heating zone, we propose a multi heating zone temperature prediction framework based on transfer learning (TL) [22]. TL uses the knowledge learned from a problem to solve a related but different problem, and it has been widely used in text classification [23], image recognition [24], and other tasks [25]. Unfortunately, prior to this, there have been no studies applying TL to temperature prediction in multiple heating zones. Therefore, we propose the deep transfer learning based on temporal convolutional networks for heating furnace temperature prediction for the first time.

According to the disadvantages of the neural network, this paper chooses the suitable heating zone as the source data. Then, we redesign and optimize TCN as the source domain model, fine-tuning the high-level weight of the source model through the data generated by GAN to complete the transfer of knowledge. The main contributions of this paper are summarized as follows:

- This paper describes the first time that transfer learning is used to solve the problem of temperature prediction in multiple heating zones of the same furnace. First, we use the generative adversarial loss for domain adaptation, and we then use the fine-tuning method to complete the target domain task. The framework proposed in this paper can obviously improve the prediction accuracy.
- Combining with the auto-correlation function and maximum mean discrepancy (MMD), a sliding window size selection method in the context of transfer learning is first proposed, which provides a novel idea for window size selection.
- We propose a weight initialization method for neural networks based on transfer learning in this paper.
- It is the first time that transfer learning is used to solve the problem of the neural network having no extrapolation.
- We optimize the structure and parameters of TCN to improve its prediction performance for time series.
- Through many experiments, we provide the consistent results of 10 different heating zones, which prove that the TCN optimization method and the transfer learning framework proposed in this paper are the most advanced methods for multiple heating zone prediction.

The rest of this paper is organized as follows. Section 2 introduces the theoretical background of the framework, including TCN, GAN, and TL. Section 3 introduces the structure of the framework and the related preparations based on the framework. In Section 4, the framework is described in detail, and nine target datasets are used for experimental research. Conclusions and future work are presented in Section 5.

## 2. Related Work

From the above analysis, we can see that the heating process has the characteristics of hysteresis, parameter diversity, and multi-objective prediction, so we need to predict the temperature change trend in advance. Compared with the classical LSTM network, we believe that TCN has better performance in furnace temperature prediction. Due to the sharing of the parameters in each heating zone, we use transfer learning to solve the multi heating zone temperature prediction task. Therefore, this part mainly introduces the related knowledge of TCN and transfer learning, as well as the improvements we have made to them, so as to predict the furnace temperature more accurately.

### 2.1. Temporal Convolutional Network

As mentioned earlier, we need to establish a billet temperature prediction model to predict the billet temperature in advance. Therefore, a mapping function as shown in Equation (1) needs to be established in order to predict billet temperature; it can predict the output *y* at time *t* only by input data $\left\{x_{1},x_{2},\ldots x_{t-1}\right\}$, without relying on future input data $\left\{x_{t},x_{t+1},\ldots x_{T}\right\}$. TCNs introduce causal convolution to deal with such sequence problems without information leakage.

(1) $${\hat{y}}_{t}=f(x_{0},x_{1},\ldots x_{t-1}).$$

Our goal is to find the mapping function f(⋅) that minimizes the error between the predicted result and the actual value.

**Figure 1:**
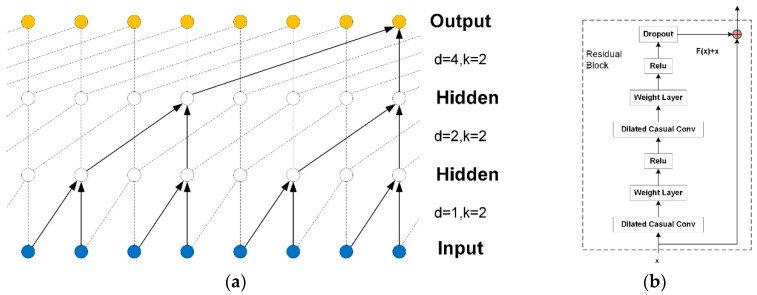
(**a**) Architectural elements in a temporal convolutional network (TCN); (**b**) residual structure of TCN.

After the above analysis, the prediction model of furnace temperature can be regarded as a series modeling problem based on the time data. Long-term and effective history data are required in order to minimize the errors between predicted and observed values. For the TCN prediction model of furnace temperature, the capturing history information is limited by the size of the convolution kernel. Using dilated convolution makes it possible for the network to obtain a large receptive field with a relatively small number of layers. More formally, for a 1 − *D* sequence input $x\in R^{n}$ and a filter $f:\left\{0,1,\ldots k-1\right\}\to R^{n}$, the furnace temperature F at time *t* is defined as follows:

(2) $$F(t)=(x_{d}*f)(t)=\sum_{i=0}^{k-1}f(i)\cdot x_{t-d\cdot i},$$

where d is the dilation factor, k is the filter size, and t−d⋅i accounts for the direction of the past. The effective history of one such layer is (k−1)⋅d. When d=1, a dilated convolution becomes a regular convolution. We provide an illustration in Figure 1a. The network’s receptive field *RF* is defined as follows:

(3) $$RF=k\cdot d_{\max}.$$

The longer the dependency that it captures, the deeper the layers that it stacks. The increase in the number of convolutional layers brings the problems of gradient disappearance, complex training, and poor fitting effects. In order to solve these problems, TCN introduces residual connection [18] to realize effective training of a deep network. Residual connection allows the network to transfer information across layers, and it contains a branch that leads out to a series of transformations, F, whose outputs are added to the input, x, of the block. A residual block consists of two layers of dilated causal convolution and nonlinear mapping. Weight norm and dropout are added to each layer to regularize the network. The residual block in the basic TCN structure is shown in Figure 1b.

Formally, we consider defining residual blocks as follows:

(4) o=Activation(F(x)+x).

In addition, 1D fully-convolutional network (FCN) [26] architecture is also used in the TCN. It accepts input of any size and uses the convolution layer to up-sample the feature map of the last convolution layer, returning it to the same size as the input and then predicting. This gives our framework the ability to handle arbitrary input length sequences.

Since we cannot directly measure the billet temperature, we need to accurately predict the furnace temperature. In this regard, we improve the structure and parameters of the TCN. Considering the diversity of heating parameters, we add a new convolution layer before the dense layer of the TCN to extract more comprehensive features. In addition, we propose a weight initialization method based on transfer learning. Details of these two improvements are presented in Section 4.

### 2.2. Transfer Learning for Time Series

As mentioned before, re-modeling in each heating zone will increase the calculation cost, and, due to a series of characteristics of industrial data and the limitation of the number of samples, the prediction error of the existing model may not meet the production requirements. Transfer learning is a novel method to solve the temperature prediction of multiple heating zones in a heating furnace; it applies the knowledge learned in the current heating zone to another different but related heating zone, making it more efficient and more accurate when completing new tasks.

Although transfer learning and deep learning have been widely used in computer vision and natural language processing, there is no complete and representative work in time series processing. The authors of [27] systematically discuss time series classification based on deep transfer learning. Knowledge transfer is accomplished by fine-tuning. Since then, transfer learning has been successfully applied to time series fields such as wind speed prediction of different wind farms [28] and life prediction of manufacturing tools [29]. However, none of the above papers introduce the domain adaptive method of time series. Inspired by this, this paper first proposes a framework that combines transfer learning and deep learning knowledge to solve the problem of multiple heating zone temperature prediction. In addition, a GAN network is used to realize domain adaption in order to maximize the similarity between the target domain and the source domain.

According to the general transfer method, the transfer learning method can be divided into instance transfer, feature transfer, relationship transfer, and model transfer [30]. Since this article studies the temperature prediction of different heating zones of the same furnace, the heating conditions of each heating zone are similar. Therefore, we propose the transfer learning framework based on model and feature. The model-based transfer learning method applies the learning model in the source domain to the target domain and then tunes it with the target domain data to form a new target model. Feature-based transfer transforms the features of the source domain and target domain to the same space through feature transformation for knowledge transfer.

### 2.3. Generative Adversarial Networks

GANs simultaneously train two models: a generative model, *G*, that captures the data distribution, and a discriminative model, *D*, that estimates the probability that a sample came from the training data rather than G [31]. The goal of *D* is to realize the two classifications of data sources: real data or fake data *G*(*z*) from a generator. The goal of *G* is to generate fake data *G*(*z*) that make *D* unable to discern the data source. In other words, *D* and *G* play the following two-player minimax game with value function *V*(*G*,*D*):

(5) $$\underset{G}{\min}\underset{D}{\max}V(D,G)=\underset{G}{\min}\underset{D}{\max}(E_{x\sim p_{data}(x)}[\log D(x)]+E_{z\sim p_{x}(z)}[\log(1-D(G(z)))]).$$

In the above equation, the mathematical meaning is divided into two parts.

1. The generator *G* is fixed, and the discriminator *D* is trained.

(6) $$\underset{D}{\max}(E_{x\sim p_{data}(x)}[\log D(x)]+E_{z\sim p_{x}(z)}[\log(1-D(G(z)))]).$$

*D* is trained to maximize the value of the above formula. The real data are divided into 1 by *D*, and the generated data are divided into 0. In the first term of the above formula, if there is a real datum that is mistakenly divided into 0, then $E_{x\sim p_{data}(x)}[\log D(x)]\to -\infty$. In the second term, if one generated datum is divided into 1, then $E_{z\sim p_{x}(z)}[\log(1-D(G(z)))]\to -\infty$.

2. The generator *G* is trained.

(7) $$\underset{G}{\min}(E_{x\sim p_{data}(x)}[\log D(x)]+E_{z\sim p_{x}(z)}[\log(1-D(G(z)))]).$$

*G* is trained to minimize the value of the above formula and make *D* unable to distinguish true and fake data. The first term of the above formula does not contain *G*, which can be ignored:

(8) $$\underset{G}{\min}(E_{z\sim p_{x}(z)}[\log(1-D(G(z)))]).$$

In the existing research, GAN is almost used to generate images, but there is little research on generating time series. In this paper, we use GAN as a feature generator to generate time series, and the generated features replace the relevant features of the target domain to maximize the similarity between the source domain and the target domain.

GAN often has the problem of pattern collapse, which is manifested in the poor results, and even after the training time is extended, it cannot be improved very much [32]. To solve this problem, after using GAN for unsupervised learning, we adopted a fine-tuning method to use the target variables of the target domain for supervised learning in order to improve the prediction accuracy of the target domain.

## 3. Data Processing and Analysis

### 3.1. Heating Process of Heating Furnace

**Figure 2:**
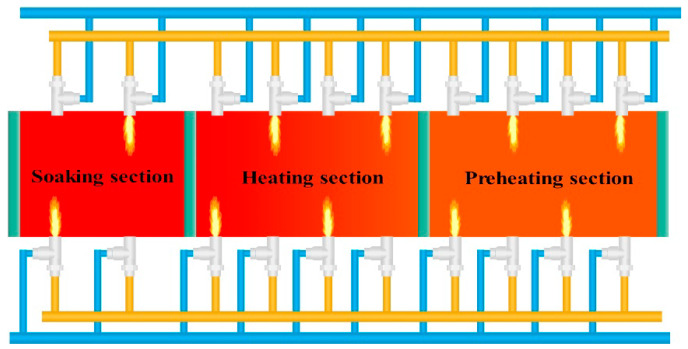
Heating process of heating furnace. The heating furnace is a three-stage heating furnace. The heating zones 1 to 4 are located in the preheating section, 5 to 8 in the heating section, and 9 and 10 in the soaking section.

The heating process of the heating furnace case studied in this paper is shown in Figure 2. The heating furnace model is a walking beam type. The heating furnace is a three-stage heating furnace, which is subdivided into a preheating section, a heating section, and a soaking section, with a total of 10 heating zones. There is a pair of burners in each zone; the odd numbered zone is for upper burn, and the even numbered zone is for lower burn. The temperature detection value is collected by different types of thermocouple sensors of the heating furnace combustion system. In order to accurately monitor the furnace temperature, the thermocouple type is B series, the material composition is PtRh-PtRh (IEC584), and the temperature monitoring range is 200–1800 °C. Finally, the sensor data collected based on fieldbus technology are stored in the distributed database.

### 3.2. The Overall Framework

**Figure 3:**
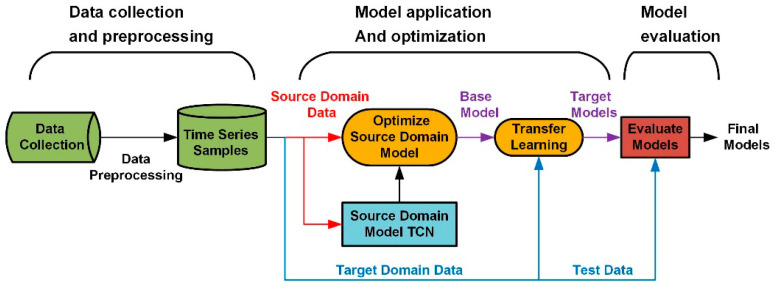
The first part preprocesses the collected original data, including the selection of relevant variables, the conversion of collected data into time series samples, the determination of sliding window size, as well as the determination of the source domain and target domain of this case. The second part trains and optimizes the structure and parameters of the TCN model according to the source domain data and then uses the target domain data to monitor and learn the basic model. In the third part, the prediction error and accuracy of the target domain model are evaluated by the target domain test and finally output the target model.

According to the actual production situation, we present the framework of this case study as shown in Figure 3. It includes the following three parts: (1) data collection and preprocessing, (2) optimization of TCN model and application of transfer learning, and (3) evaluation of target model on actual data.

Based on the framework, this section will introduce the first part of the framework in detail, including data processing, source domain determination, and sliding window modeling.

### 3.3. Data Collection and Processing

The study collected actual production data of the furnace, which has 10 heating zones in a line of 1500 mm hot-rolled broadband. The data were acquired between 10:00 on 24 January 2019 and 10:00 on 25 January 2019. The sampling frequency of each heating zone is 1/30 Hz. First two missing values are removed, so there are 2859 samples per heating zone. The control variables include 62 variables such as air pressure, oxygen flow, gas flow, nitrogen flow, valve opening, etc. Finally, the first 70% of each set of heating zone data is used as the training set and the last 30% is used as the test set.

Since these 10 heating zones are located in the same furnace, all heating zones have the same heating system. After variable selection based on prior knowledge, we take heating zone 1 as the benchmark, and the number of different variables in other heating zones and heating zone 1 is shown in Table 1. It can be seen from the table that there is high similarity between the heating zones, so we have reason to use transfer learning to complete the task of temperature prediction in each heating zone.

### 3.4. Source Domain and Target Domain

In the process of transfer learning, there is no uniform standard for the selection of source domain, which usually depends on the actual situation and prior knowledge. A superior source model can be obtained by a suitable source domain. In this way, knowledge transfer results will be more ideal.

In the course of the study, we found that the extrapolation ability of the deep learning method is not strong. This is due to the fact that a neural network can map virtually any function by adjusting its parameters according to the presented training data. The output of the neural network is unreliable for the variable space region without training data. In our case study, when the temperature range of our test set is outside the temperature range of the training set, it is difficult to predict it accurately. It can be roughly divided into two categories. Firstly, when the temperature range of the test set is outside the temperature range of the training set, it is difficult to accurately predict. Secondly, when the temperature range of the test set is within the temperature range of the training set, but the density of the test set in the training set is very low, it is also difficult to predict accurately.

**Figure 4:**
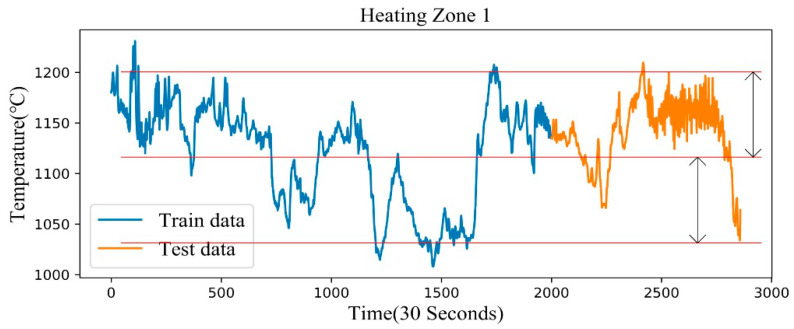
Training and test set distribution of the target variable in heating zone 1.

**Figure 5:**
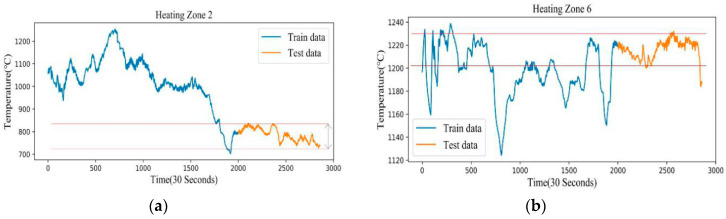
We choose two heating zones to show that the neural network has no extrapolation (**a**) Table 2. (**b**) Training and test set distribution of target variable in heating zone 6.

**Figure A1:**
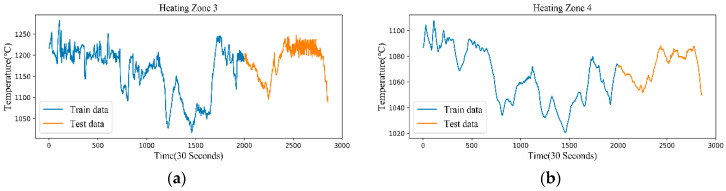

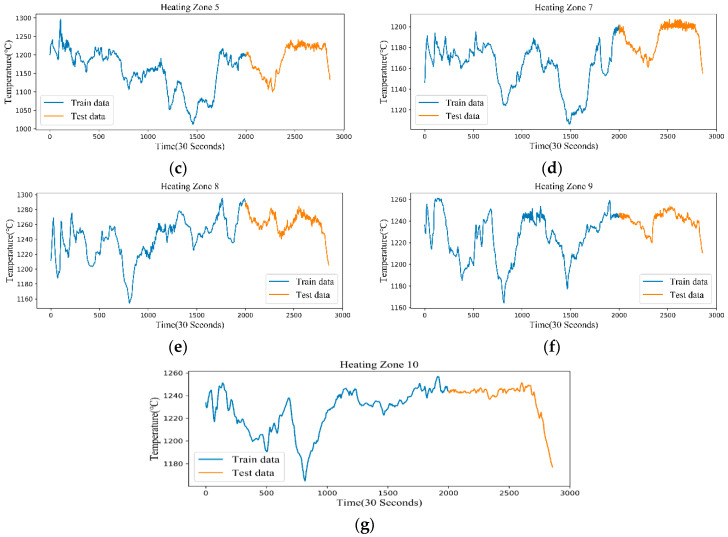
Temperature distribution of target variables in each heating zone except those mentioned in the manuscript, (**a**–**g**) shows the temperature distribution of heating zone 3–10 except heating zone 6.

In our study, the heating zone 1 is selected as the source domain according to the actual production situation and the distribution of the predicted target. Our reasons are as follows. The temperature distribution of heating zone 1 is shown in Figure 4, and it can be seen that the test set of zone 1 falls into a high-density training set. This means that the temperature value of the test set is repeatedly trained in the training set, so as to ensure that the basic model of pre-training on the source domain can achieve the optimal performance. Figure 5 shows two representative heating zones, Figure 5a shows the temperature distribution of heating zone 2, and Figure 5b shows the temperature distribution of heating zone 6. The temperature distribution of these two zones corresponds to the second case. The temperature distribution and furnace temperature control requirements for the remaining heating zones are shown in Figure A1 and Table A1 and Table A2 of Appendix A. In order to ensure good migration learning performance, we choose zone 1 as the source domain.

From Table A1 and Table A2 in Appendix A, there are four different steel brands. The processing technology of billets with the same steel brand is the same, and the heating process is similar. Therefore, we only need to select the source domain once for each steel brand, and the source domain selected for processing the same type of steel is the same. It avoids selecting the source domain every time.

### 3.5. Sliding Window Model

After data collection, preprocessing, and source domain determination, the data should be processed on the premise of retaining time sequence information. Our data format should conform to the format of TCN input for supervised learning. To achieve this, the sliding window method is commonly used.

**Figure 6:**
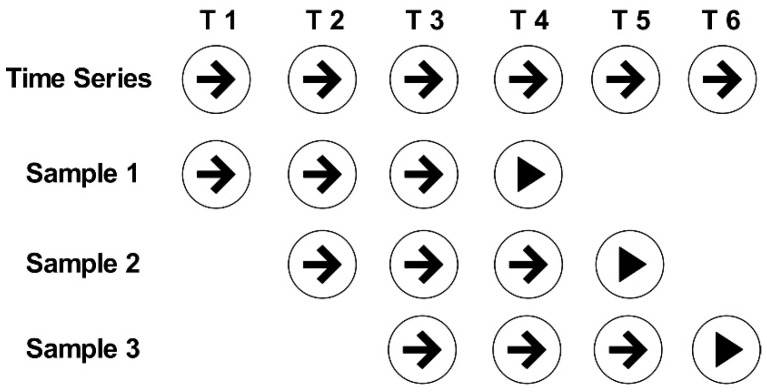
An example of constructing time series samples by sliding window.

An example of constructing time series samples using a sliding window is presented in Figure 6. We assume that there are six time-series samples in the dataset, including T1, T2... T5 and T6. If the window size ∆t = 3 for sample 1, it has T1, T2, and T3 as its features and T4 as its label. Similarly, sample 2 and sample 3 are given. The size of the window will affect the number of time series samples and the features in the sample. Therefore, it is necessary to define an optimal window size to ensure that our model has an optimal prediction result.

**Figure 7:**
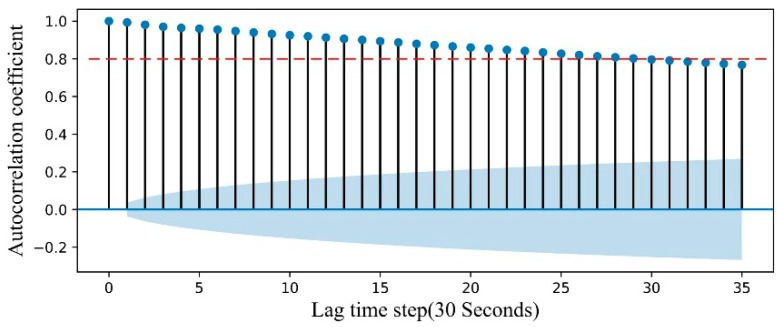
Auto-correlation within different sliding window sizes.

In this study, we use transfer learning to predict the temperature of multiple heating zones; therefore, it is necessary to consider both the source domain prediction accuracy and the target domain prediction accuracy. Considering the effectiveness of knowledge transfer, the method combining auto-correlation function [33] and maximum mean discrepancy (MMD) [34] is proposed for the first time to determine the sliding window size in the context of transfer learning. The auto-correlation coefficient is used to determine the temporal correlation between the time series data themselves. A larger value of the correlation coefficient means a time correlation and a stronger lagged effect. In this paper, the auto-correlation coefficient is defined as follows:

(9) $$\rho_{t,t+\Delta t}=\frac{Cov(y(t),y(t+\Delta t))}{\sigma_{y(t)}\sigma_{y(t+\Delta t)}},$$

where Cov(⋅) represents the covariance, and σ(⋅) represents the variance. It represents the correlation of a time series at any t time and t+Δt time. The calculation result of the target variable in the source domain is shown in Figure 7.

In general, an auto-correlation coefficient greater than 0.8 represents a high correlation. Figure 7 shows that, when the auto-correlation coefficient is greater than 0.8, the lag time step is 28. Therefore, we narrow the sliding window size Δt to (1, 28) for the next step.

The initial sliding window size is given by the source domain data, and we also need to consider the prediction accuracy of the target domain. Our goal is to apply knowledge learned from the source domain to different but related target domains. The transfer effect is best when the difference in data distribution between the two domains is minimal. *MMD* can be used to measure the difference in data distribution between two domains. Therefore, we propose to use *MMD* to determine the final sliding window size. *MMD* was first proposed for the two-sample test problem to determine whether the two distributions *p* and *q* are the same [35]. We assume that *X* and *Y* are two datasets obtained by independent and identically distributed sampling from *p* and *q*. The squared distance of the two distributions is defined as follows:

(10) $$MMD(F,X,Y):=\underset{f\in F}{\sup}(\frac{1}{n}\sum_{i=1}^{n}f(x_{i})-\frac{1}{m}\sum_{i=1}^{m}f(y_{i})),$$

where f is a continuous function on the sample space, and *F* is a given set of functions. When *F* is the unit ball on the kernel Hilbert space, *MMD* can quickly converge. Therefore, *MMD* can be defined as follows:

(11) $$MMD^{2}(F,X,Y)=\left|\right|\frac{1}{n}\sum_{i=1}^{n}f(x_{i})-\frac{1}{m}\sum_{i=1}^{m}f(y_{i}){\left|\right|}_{H}^{2},$$

where f(⋅) represents a mapping function, and H represents that this distance is measured by f(⋅) mapping the data onto the reproducing kernel Hilbert space (RKHS) [36].

Since the mapping function cannot be solved directly in high-dimensional space, we use Gaussian kernel function *k* to skip the solution of mapping function. The final *MMD* calculation formula is defined as follows:

(12) $$MMD(X,Y)=\left|\right|\frac{1}{n^{2}}\sum_{i,j=1}^{n}k(x_{i},x_{j})-\frac{2}{nm}\sum_{i,j=1}^{n,m}k(x_{i},y_{j})+\frac{1}{m^{2}}\sum_{i,j=1}^{m}k(y_{i},y_{j}){\left|\right|}_{H}^{}.$$

Based on the above theoretical analysis, the specific process by which *MMD* is used to determine the sliding window size is as follows: (1) solve the *MMD* values of the source and target domains at a sliding window size, (2) traverse all-time series samples under this sliding window size and find the *MMD* mean of all samples under this sliding window size, and (3) traverse the range of sliding window sizes determined by the auto-correlation function, traversing all sliding window size values in this range. As shown in Figure 6 above, when the sliding window size is 3, the *MMD* scores of the source domain and the target domain in sample 1, the *MMD* scores of the source domain in sample 2, and the *MMD* scores of sample 3 are calculated. Then, the *MMD* scores of these three samples are averaged. When the sliding window size is other values, the same method is used to obtain the *MMD* score.

**Figure 8:**
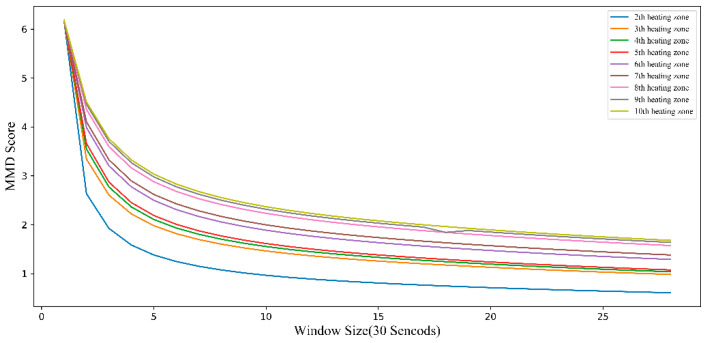
Maximum mean discrepancy *(MMD)* scores of source domain and target domain under different sliding window sizes. This score represents the similarity measurement between the rest of the zones and zone 1. The figure in the upper right corner refers to the *MMD* scores of zone 2 and zone 1, and the *MMD* scores of zone 3 and zone 1, all the way to zone 10.

In our study, the size range of sliding window determined by the autocorrelation function is (1, 28), so we obtain an *MMD* score in this range, as shown in Figure 8. It can be seen from the figure that the larger the sliding window size, the smaller the *MMD* score. When the sliding window size is 28, the *MMD* score is the lowest, and the similarity between source domain and target domain is the highest, so the transfer result is the best. Combining the auto-correlation function and the calculation results of *MMD*, the sliding window size of this study was finally determined to be 28.

## 4. Methodology and Results Analysis

### 4.1. Technical Details

All implementations of the transfer learning framework proposed in this article were performed on personal desktops with Core i7-8700 (3.20 GHz), 16 GB RAM, NVIDIA GTX1060 6 GB, and 64-bit operating system. The operating system was Windows10 Professional and Python 3.6 with tensorflow and keras. Since the number of samples was not very large, all algorithms were run in a CPU environment.

### 4.2. TCN for Furnace Temperature Prediction

Since the heating process was continuous, TCN was used as the prediction model in this paper. In this study, we optimized the structure and parameters of the TCN model proposed in [19]. The experimental results show that our improved TCN model has better performance in time series prediction.

**Figure 9:**
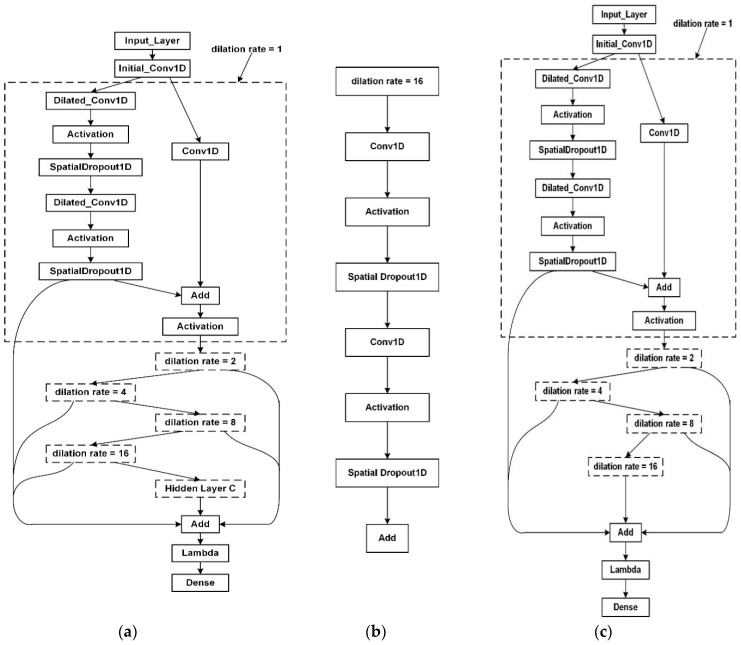
The TCN structure proposed in this paper and the initial TCN structure proposed in [19]. (**a**) shows the TCN structure used in this paper; hidden layer C is added to the TCN structure. (**b**) is the structure of hidden layer C. (**c**) is the initial TCN structure proposed in [19].

We set the size of 1−*D*, convolution kernel size k=2, as mentioned before; the receptive field of TCN was expressed as $k\cdot d_{\max}$. We entered a sliding window size of 28. Therefore, the maximum dilation rate $d_{\max}$ of TCN was $d_{\max}\geq 16$. The dilation rate in this paper was [1,2,4,8,16]. The structure of the TCN is shown in Figure 9a. After setting the hidden layer D = 16, we added the hidden layer C to optimize the TCN structure. As shown in Figure 9b, in this layer, we did not use the residual structure, and we used two convolution layers to extract features. This hidden layer can extract more effective features to get better results. The proposed TCN structure includes input layer, initial convolution layer, 5 residual block structures, hidden layer C, and finally a fully connected layer. It has 56 layers in total. We choose Keras [37] as our deep learning framework for training the TCN model. Considering the effect of the rectified linear unit (RELU) activation function on the output, and in order to ensure the consistency of the input and output variances, he-normal [38] is selected as the method of weight initialization. After simple tuning of TCN parameters, the number of convolution kernels was 64 per layer, and the value of dropout rate was 0. When we trained the TCN model, we set the value of epoch to 100 and selected Adam [39] as the optimizer to adapt to the learning rate.

In order to test this network structure, we first used the source domain dataset to compare its prediction performance with several other commonly used prediction models, which included LSTM, gated recurrent units (GRU) [40], convolutional neural network-LSTM (CNN-LSTM), and bi-directional LSTM (BiLSTM) [41]. LSTM is an improved recurrent neural network (RNN) that exhibits excellent performance in processing time series data. GRU simplifies the LSTM structure for faster training. BiLSTM is based on LSTM and can learn from long-term dependencies in both forward and backward data of the time series. CNN-LSTM is a combination of convolutional neural networks and LSTM, which performs better in most studies. The detailed information of these networks is shown in Table 2, where the parameters are determined by grid search.

In order to measure the prediction results of each model, we choose the root mean square error (*RMSE*) and the mean absolute error (*MAE*) as the main evaluation indicators of the regression prediction. The definitions of RMSE and MAE are as follows:

(13) $$RMSE=\sqrt{\frac{1}{n}{\sum_{i=1}^{n}\left(y_{i}-{\hat{y}}_{i}\right)}^{2}},$$

(14) $$MAE=\frac{1}{n}\sum_{i=1}^{n}\left|(y_{i}-{\hat{y}}_{i})\right|.$$

where *n* represents the number of samples, $y_{i}$ represents the observation of the *i*th sample, and ${\hat{y}}_{i}$ represents the predicted value of the *i*th sample. Lower *RMSE* or *MAE* is better than a higher one. The smaller the *RMSE* and *MAE* values, the higher the prediction accuracy, and the better the model performance.

As mentioned earlier, the data of heating zone 1 were used as the source domain data: the first 70% of the data were used as the training set, and the remaining 30% of the data were used as the test set. The mean values of *RMSE* and *MAE* after multiple training procedures of these models are shown in Table 3. From the data shown in the table, we can easily see that our proposed TCN model performed best in this case. Although the reported mixed model CNN-LSTM is a relatively advanced sequence processing model, this case does not support this statement. In addition, GRU and BiLSTM perform better than LSTM.

As mentioned earlier, we redesigned the structure of the TCN. As shown in Figure 9a, in the last Add layer, six sources of information were added as hidden layers with dilation rates of 1, 2, 4, 8, and 16 and hidden layer C. Among them, the hidden layer C was added in order to extract the features of the previous hidden layers after the hidden layer with the dilation rate of 16. The initial TCN model is shown in Figure 9c, and the final add layer adds the information of five hidden layers with dilation rates of 1, 2, 4, 8, and 16. Therefore, our improved TCN can extract more comprehensive features and obtain better results.

Therefore, we use the university of California, Irvine (UCI) open source dataset Beijing PM2.5 data for experimental verification. The dataset is described in Table 4. The time period of the data is from 1 January 2010 to 31 December 2014, with a one-hour interval between each datum. We used the attributes measured in the past 12 h, including dew point, temperature, pressure, and so on, to predict the PM2.5 concentration in the next hour. We took the data of the first 3.5 years as the training set and the remaining data as the test set. Since only 12 h of historical data were needed, we set the expansion rate to $d_{\max}=8$, and the other parameters were the same as the furnace temperature prediction. Table 5 shows the score in comparison between the initial TCN and the improved TCN. The experimental results show that the improved TCN is better than the initial TCN in time series prediction.

**Figure 10:**
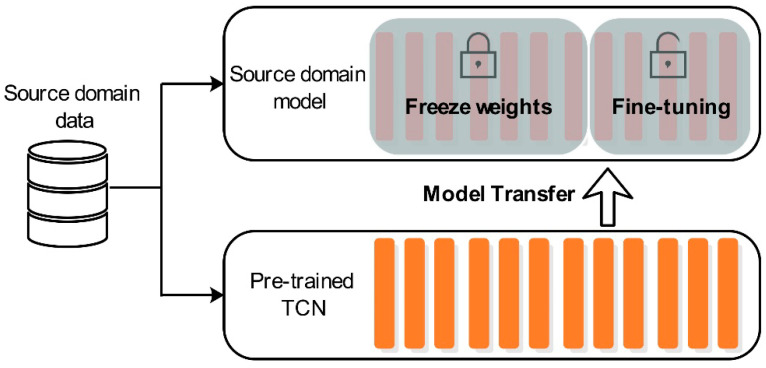
Using self-transfer learning as a method of neural network initialization. Some of the hidden layers of pre-training were frozen, and then the unfrozen layers were updated with their own training set.

In addition to the improvement of the TCN structure, we used the idea of transfer learning to optimize the TCN parameters. We called this a method of neural network weight initialization. The specific process of optimization is to freeze the shallow weight of TCN and then use the training set to update the parameters of the unfrozen high-level features, which we call self-TL-TCN. First of all, we used the training set in the source domain (heating zone 1) to train a preliminary TCN model. The TCN structure proposed in this paper contains 56 hidden layers, but only the weight and bias of the convolution layer need to be updated. We froze from layer 20 of the pre-trained TCN—that is, from the hidden layer with an expansion rate of 4. Finally, we used the training set in the source domain to update the parameters of the unfrozen layer to complete the fine-tuning. The reasons for this are as follows: (1) the optimized model is given more appropriate initial weight compared to the he-normal method, (2) the convolution layer with high dilation rate of the preliminary TCN model may cause information loss. The training process is shown in Figure 10.

**Figure 11:**
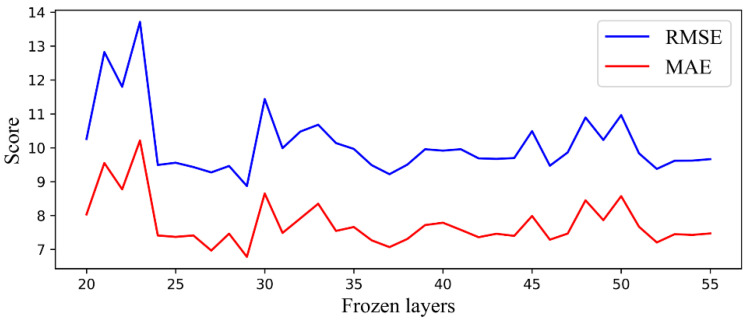
Performance of TCN under different frozen layers.

The performance of the TCN after parameter initialization under different freezing layers is shown in Figure 11. It can be seen that, when the number of freezing layers is 29, we obtained the lowest RMSE and MAE scores. Therefore, when optimizing the preliminary TCN model, the best prediction performance can be achieved by freezing the first 29 layers and fine-tuning the parameters of the upper layer. The self-TL-TCN model after two optimizations of structure and parameters is the final source domain model in this study.

**Figure 12:**
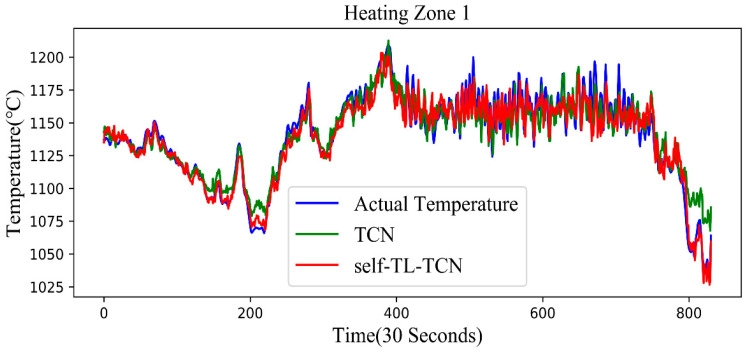
Prediction results before and after TCN optimization.

After determining the optimal number of freezing layers, we used the test set of the source domain to evaluate. Figure 12 shows the prediction results of the TCN model before and after optimization. It can be seen from the figure that the prediction accuracy of self-TL-TCN proposed by us is higher.

**Figure 13:**
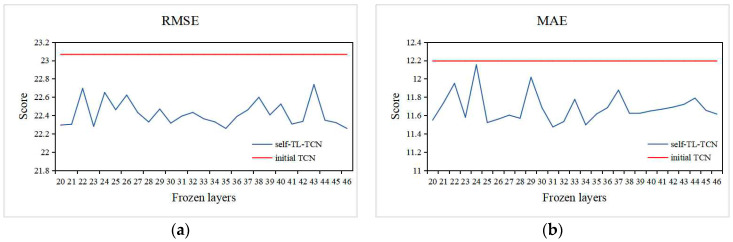
We compare the TCN network after two improvements with the TCN proposed in [19], (**a**) is the RMSE score under different frozen layers, and (**b**) is the MAE score under different frozen layers.

In order to verify the effectiveness of the proposed network, we also used the Beijing PM2.5 dataset to verify. Since we used the data from the first 12 h as the historical data, the TCN network was layer 46, and we chose to freeze from layer 20. Figure 13 shows the scores of self and initial TCN under different freezing layers. The comparison results between the optimized TCN model and the original TCN model are shown in Table 6. As can be seen from the figure, the method using self-transfer learning as initialization parameter can achieve better prediction results.

### 4.3. Transfer Learning for Furnace Temperature Prediction

As mentioned before, a heating furnace system has multiple heating zones. We need to accurately predict the temperature of each heating zone, but because the heating furnace is a complex controlled object, its temperature curve is nonlinear and unstable, among other characteristics; there will be some heating zone temperatures which are difficult to accurately predict. For example, for a heating zone such as zone 2, the neural network is ineffective. In addition, because all heating zones are in the same furnace, the heating process is similar. As shown in Table 1, the control variables of each heating zone are very similar, and, at most, only nine variables are different. Therefore, we believe that the knowledge learned by the neural network in each heating zone is also similar. Therefore, we can transfer the knowledge learned by the neural network in a heating zone that can accurately predict the temperature to the remaining heating zones. There are 10 heating zones in this case. If we build a model for each heating zone, different heating zones may have different neural network models, which will undoubtedly increase the calculation cost. This is also one of the reasons that we used transfer learning.

Since our goal was to build a model that can solve different tasks of the same industrial equipment, the data collected by industrial sensors can be divided into two types. The first is that different tasks share a control system—that is, the same variable controls different tasks, such as the prediction of water content and temperature at the outlet of the dryer in the tobacco factory. The control variables of these two tasks are the same. The other is that different control systems control different task requirements. For the first kind of sensor data, because the characteristics of each domain are the same, the fine-tuning method was used to complete the knowledge transfer. For the second kind of sensor data, we used the generative adversarial loss to complete the domain adaptation. We took the sensor data of the heating furnace as an example to verify the performance of the two methods. This was also the first time that transfer learning has been applied to the prediction of furnace temperature.

**Figure 14:**
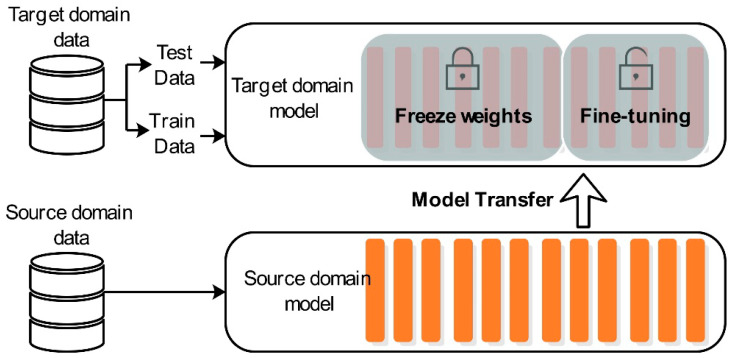
Using the target domain training set to fine-tune the unfrozen layer of the source model to obtain a new target model TL-TCN and then using the target domain test set to test the target model.

As mentioned before, because each heating zone is located in the same furnace, there is high similarity between the target domain and the source domain. For example, only one control variable is different in zone 1 and zone 2, and the difference between zone 1 and zone 10 is the largest, but only nine variables are different. Each heating zone has 62 control variables. Therefore, we used the fine-tuning method shown in Figure 14 to complete the transfer.

Through traversing all hidden layers of the network to determine the optimal number of frozen layers in each heating zone, the optimal number of frozen layers in each heating zone was calculated, as shown in Table 7. The reason that the number of freezing layers in the table is small is that the neural network mentioned before has no extrapolation—that is, the distribution of the test set and training set is quite different. This means that more parameters need to be updated for better results. The temperature distribution of each zone is shown in Appendix A, Figure A1.

**Figure 15:**
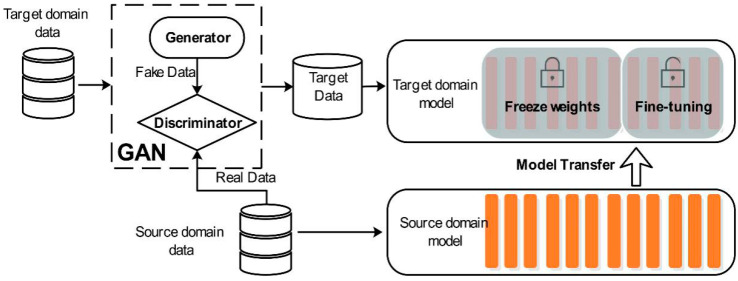
The domain adaptation is achieved by the generative adversarial network, and the target model is fine-tuned by the target variable of the target domain.

For the different features of the source domain and the target domain, we used the generative adversarial network to align the features, as shown in Figure 15. We used the source domain data as the real data input discriminator. The discriminator uses three full connection layers as two classification layers to judge the data source. To prevent overfitting, dropout layers were added between the second and third layers. The first two layers use RELU as the loss function, and the third layer uses Sigmoid. In this case, the generator in GAN was used as the feature extractor, and we used the 1−*D* convolution to extract the target domain features. The generator generates time series features with higher similarity to the source domain in order to replace the original target domain features. When the discriminator cannot judge whether the data is generator data or real data, this shows that the features extracted by the generator have high similarity with the features of the source domain. In addition, this case is supervised learning. In order to further improve the prediction accuracy, we need to use the target variable of the target domain to fine-tune the target model after using GAN as the feature alignment. Therefore, we added a fine-tuning strategy after GAN to generate the final target model.

**Figure 16:**
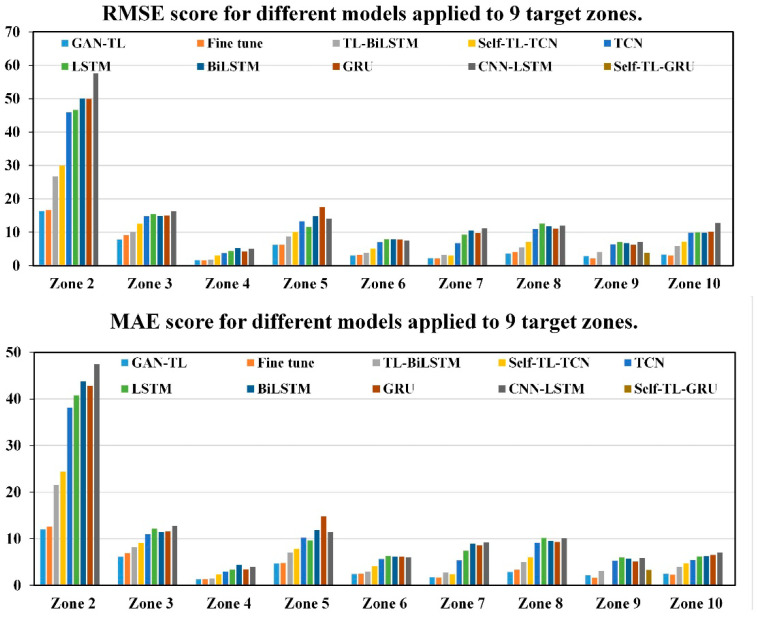
RMSE and MAE scores of different models applied to nine target zones.

We established TCN, LSTM, BiLSTM, GRU, and CNN-LSTM models on nine target domains. We chose the best performing model per target domain to optimize. The optimization method uses self-transfer learning to change the network weight initialization. In addition, the transfer learning method was based on the BiLSTM proposed by Jun Ma et al. [30], which greatly improved the air quality prediction results. In our paper, first of all, the initial BiLSTM was established on the source domain and was optimized based on the self-transfer to obtain self-TL-BiLSTM. Then, the target domain model TL-BiLSTM was established based on transfer learning. We compared the prediction results of these models with the two methods proposed in this paper. Table 8 shows the RMSE scores of each model applied to nine target domains. Table 9 shows the MAE scores. The last column of the two tables is obtained by comparing these two methods with the best-performing model without knowledge transfer. Figure 16 is two histograms of RMSE and MAE scores for these models applied to nine target heating zones.

The following information can be obtained from these two tables and histograms:

- The performance of the TCN model is better than the variant of the RNN in almost all zones, and the GRU is better than TCN only in heating zone 9.
- The self-TL-TCN proposed by us has better performance than the common TCN model; the self-TL-GRU also has better performance than the common GRU model. This means that network performance can be improved by changing the initial weight of the network based on the migration learning idea.
- Two transfer learning frameworks proposed by us can effectively solve the problem of large prediction error in some heating zones, which greatly reduces the prediction error. Zones 10 and 9 have the largest error reductions, with RMSE reduced by 57.38% and 43.97% and MAE reduced by 51.63% and 50.59%. TL-BiLSTM also shows better performance than models without knowledge transfer in each zone but worse performance than our models.

In addition, the reason that the fine-tuning method in zone 4, zone 7, zone 9, and zone 10 performs better is because the original target data are more similar to the source domain feature than the new feature generated. Table 10 shows the similarity between the generated features and the source domain, as well as the similarity between the original features and the source domain. We use the Pearson coefficient to measure the similarity. We only measure the similarity between the source domain and the target domain. It can be seen that the similarity between the original data of the above four regions and the source domain is higher. Of course, both fine-tuning and GAN-TL have better performance than no knowledge transfer. That is to say, fine-tuning is a good solution when the time series of the same feature is transferred. When the time series of different features are transferred, the GAN-TL method that we proposed is also a suitable solution.

**Figure 17:**
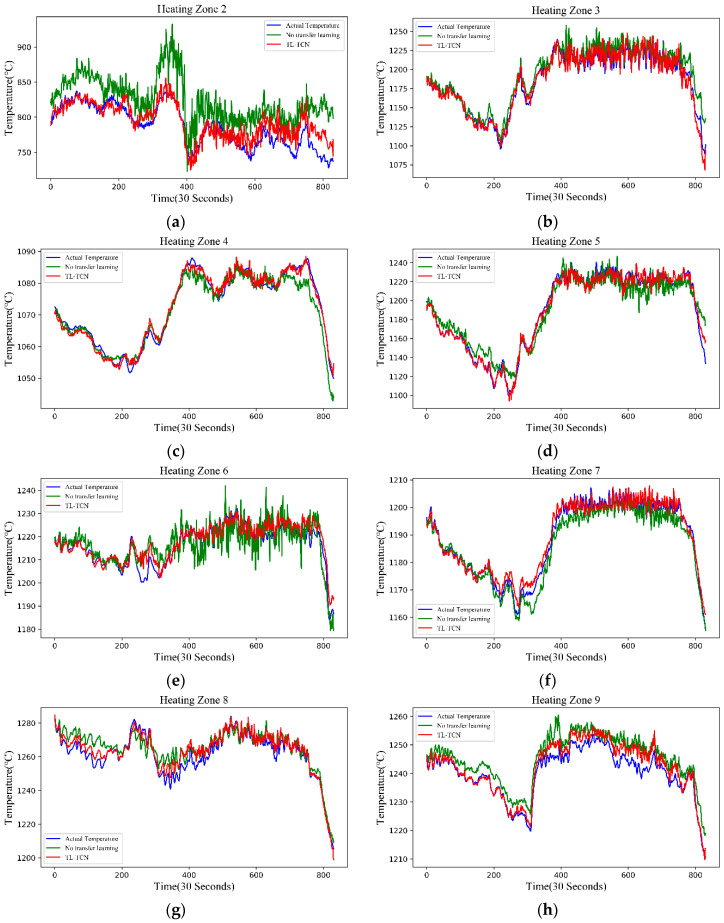

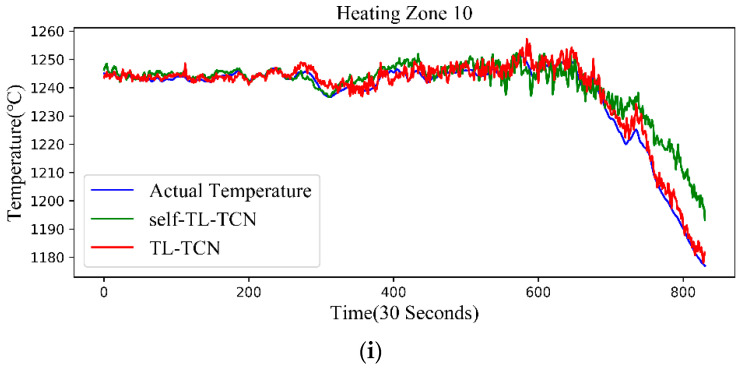
No transfer learning means that we do not use transfer learning to complete the prediction, but we use self-transfer learning to change the initial weight of the neural network. TL-TCN refers to the method with better results in fine-tuning and GAN-TCN, (**a**–**i**) shows the comparison of prediction results of heating zone 2–10.

Figure 17 shows the comparison of the prediction results based on transfer learning of all target domains with the prediction results without knowledge transfer. These prediction results prove that the neural network that we mentioned before has no shortcomings in terms of extrapolation. In other words, the data with a large difference between the test set and training set cannot produce good prediction results, such as in zone 2 and zone 6. However, we can solve this problem through transfer learning. This is the first time that we propose to solve the non-extrapolation problem of the neural network by using transfer learning.

In order to measure the performance of the proposed transfer learning framework more comprehensively, the complexity of the model is calculated. The complexity of the convolutional network is defined as follows:

(15) $$Complexity∼O(\sum_{l=1}^{D}K_{l}^{2}\cdot C_{l-1}\cdot C_{l}).$$

where *D* represents the number of convolutional layers, *l* represents the *l*-th convolutional layer, *K* represents the size of the convolution kernel, *Stride* represents the step length, $C_{l}$ represents the number of output channels. In this paper, each convolution layer has 64 convolution kernels. Since this is one-dimensional convolution, the input dimension is 62 and the size of the convolution kernel is 62 × 2.

This paper takes LSTM as an example to illustrate the computational complexity of recurrent neural networks. The complexity is defined as follows:

(16) $$Complexity∼O(\sum_{l=1}^{D}4\cdot {((X}_{l}{+H}_{l}{)H}_{l}+H_{l})).$$

where *D* represents the number of hidden layers, *l* represents the *l* th hidden layer, *X* represents the input dimension, and *H* epresents for hidden layer size.

Table 11 shows the calculation results of the complexity of each model based on the above two formulas. At the same time, we calculated the running time of each model on the same computer configuration, as shown in Table 12.

From the above two tables, and combined with the prediction results, we can see that the framework proposed in this paper has the best performance, whether in terms of runtime or prediction results. On one hand, the time prediction framework based on TCN is a stack of convolutional layers, and the convolution kernels of each layer are shared, so the calculation time will be greatly accelerated. On the other hand, the target model only needs to train unfrozen parameters, which will greatly reduce the number of parameters.

In order to verify the idea that using transfer learning under similar tasks will produce better results than not using transfer learning, we also used the aforementioned open source data from Beijing PM2.5 for experimental verification. We conducted the hourly interval prediction before. However, in the context of large time resolutions such as days and weeks, it is difficult to produce high-precision prediction methods. Therefore, we used the research methods in this paper to transfer the learned knowledge from a smaller time resolution to a larger time resolution. That is to say, we transferred the knowledge that we learned at hourly intervals to predicting air quality at daily intervals. First, we re-sampled the original data at daily intervals to form the target domain data. Since we only re-sampled the original data with expanded frequency, we used the fine-tuning method for knowledge transfer. The grid search determined that the prediction of the target domain was the best when the source domain model froze the first 23 layers. Table 13 shows the score by comparison of each model. It can be seen from the above table that the method proposed in this paper can improve the prediction accuracy of PM2.5 concentration at large time resolutions. Compared with no transfer learning, transfer learning achieves better results.

In order to prove the rationality of source domain selection and the shortcomings of our neural network without extrapolation. Combined with the temperature distribution diagram of Figure A1 in Appendix A, we chose heating zone 3, with uniform temperature distribution, as the source region. Compared with other target regions, the distribution of the test set and training set in heating zone 3 was more uniform, but it was not as reasonable as that in heating zone 1. We used the fine-tuning method to transfer knowledge. Table 14 and Table 15 show the prediction results of the target domain when zone 3 is the source domain.

Table 16 is a comparison of transfer results of zone 3 as source domain and zone 1 as source domain. From Table 14, Table 15 and Table 16, it can be seen that, compared with zone 3 as the source domain, when zone 1 is the source domain, the scores of other heating zones are significantly better, except for the RMSE of zone 2. Even when the knowledge learned by the neural network is transferred from zone 3 to zone 9, there is a negative transfer phenomenon. Therefore, this experiment verifies the rationality of our source domain selection.

For industrial sensor data, through this experiment, we can establish a source selection standard. When the data distribution of the test set is outside the data distribution of the training set, or the difference between the data distribution of the test set and the data distribution of the training set is large, because the neural network has no extrapolation, the data as the source domain data may have a negative transfer phenomenon. The modified standard is not only applicable to the data of the heating furnace but also to other sensor data and could even be extended to more applications.

### 4.4. Discussion on Whether the Framework Is Overfitted

To verify the question of whether the presented transfer learning framework is “overfitted” for a concrete situation, we conducted the following experiments measuring the following three factors: (1) transfer between different pieces of equipment at the same time, (2) transfer between the same pieces of equipment at different times, and (3) transfer between different pieces of equipment at different times.

First of all, we conducted knowledge transfer of different equipment at the same time. We applied this framework to transfer between different pieces of equipment. We called the above heating furnace heating furnace 1, and we transferred the knowledge learned in zone 1 of heating furnace 1 to heating furnace 2. The sensor data of the two heating furnaces were collected at the same time. We selected three heating zones from the preheating section, heating section, and soaking section of furnace 2, namely zone 1, zone 5, and zone 10, to verify the transfer results. Table 17 shows the comparison between the proposed TL-TCN framework and the existing model.

It can be concluded from the above table that the prediction results based on TL-TCN are greatly improved compared to those obtained without transfer learning. It proves the reliability of the proposed framework to transfer between different devices. In addition, we selected three heating zones in the three heating sections of the heating furnace 2 for knowledge transfer, which also proves that the TL-TCN-based framework can transfer the knowledge learned from one source domain to any heating zone of different heating furnaces.

Secondly, we conducted knowledge transfer between the same pieces of equipment at different times. The previous data were collected from 10:00 on 24 January to 10:00 on 25 January 2019. We transferred the knowledge that they learned to the data collected from 0:00 on 24 February to 0:00 on 25 February 2019. There was an interval of one month between the two datasets. The data were collected by heating furnace 1. The source domain was still heating zone 1, and the target domain was also heating zone 1, heating zone 5, and heating zone 10. Table 18 shows the scores after knowledge transfer.

It can be seen from the above table that the proposed framework can be applied to the transfer of the same equipment at different times. Moreover, the source domain model that we used has not been recalibrated but only fine-tuned with historical data from the time before the target data were acquired on 24 February. This experiment has proven that the knowledge learned by the proposed framework can be applied to data acquired one month later.

Finally, we transferred the knowledge learned in the source domain of this article to the data of different equipment at different times. The target domain data were collected from furnace 2 from 24 February at 0:00 to 25 February at 0:00. We also selected zone 1, zone 5, and zone 10 as the target domains. Table 19 shows the prediction scores of each model.

It can be seen from the table that the proposed framework can be applied to the transfer between different pieces of equipment at different times, and we did not recalibrate the source domain model but only used the target domain data to fine-tune it. This experiment has proven that the knowledge learned by the proposed framework can be applied to data acquired one month later. However, it can be concluded from the above three different experiments that the results of knowledge transfer between different equipment and different times have not improved much. This is also the main direction of future research.

## 5. Conclusions

In conclusion, we have proposed two TL-TCN frameworks to solve the temperature prediction problem of multiple heating zones in the furnace. Experimental results show that the framework proposed in this paper has magnificent performance in 10 different heating zones. Based on the similar heating processes in different heating zones and the background of sharing most of the control variables, this paper proposes a transfer learning framework. First, the source zone is selected reasonably according to the temperature distribution, and then the knowledge learned in the source zone is transferred to the remaining heating zones. The transfer learning framework in this paper is equivalent to expanding the training set, avoiding the extrapolation prediction of neural network, and solving the problem of inaccurate prediction caused by unstable temperature distribution of heating furnace. Fine-tuning can solve the different needs of the same control variables in the same equipment. GAN-TL can solve different needs with different control variables. For the unstable and nonlinear industrial sensor data, taking the sensor data of the heating furnace as an example, our transfer learning framework provides a new way to solve the problem of the neural network without extrapolation. In addition, our neural network weight initialization method based on self-transfer learning also significantly improves the prediction performance of the network. However, when the two domains are highly similar, it is necessary to further improve the feature extraction capability of GAN in the GAN-TL framework. In future work, GAN needs to be further optimized in order to improve its performance. In addition, our future work will also focus on solutions for multiple source domains, which is also a common phenomenon in industrial systems.

## Figures and Tables

**Table 1:** The number of different variables in the remaining zones and zone 1.

Heating zone	2	3	4	5	6	7	8	9	10
Number of different variables	1	2	3	4	5	6	7	8	9

**Table 2:** Parameters of the remaining neural networks for comparison with TCN.

Parameters	CNN-LSTM	LSTM	BiLSTM	GRU
Neurons	364	128	256	256
Batch Size	64	64	64	72
Epochs	100	100	100	70

**Table 3:** Different models for prediction of heating zone 1.

	CNN-LSTM	LSTM	BiLSTM	GRU	TCN
RMSE	18.067	18.318	17.684	13.428	11.362
MAE	14.715	13.938	13.807	10.218	8.797

**Table 4:** Different models for prediction of heating zone 1.

Dataset Characteristics	Number of Instances	Attribute Characteristics	Number of Attributes
Multivariate, Time Series	43,824	Integer, Real	13 ^1^

^1^ The 13 attributes are as follows. No: row number; year: year of data in this row; month: month of data in this row; day: day of data in this row; hour: hour of data in this row; pm 2.5: PM 2.5 concentration (ug/m^3); DEWP: dew point (â„ƒ); TEMP: temperature (â„ƒ); PRES: pressure (hPa); cbwd: combined wind direction; Iws: cumulated wind speed (m/s); Is: cumulated hours of snow; Ir: cumulated hours of rain.

**Table 5:** Performance of TCN before and after structure improvement.

Dataset		TCN	Improved TCN
Heating furnace	RMSE	12.509	11.362
	MAE	9.596	8.797
Beijing PM 2.5	RMSE	23.065	22.399
	MAE	12.194	11.738

**Table 6:** Performance of TCN before and after structure and parameter.

Dataset		TCN	Self-TL-TCN	Improvement
Heating furnace	RMSE	12.509	8.870	29.09%
	MAE	9.596	6.781	29.33%
Beijing PM 2.5	RMSE	23.065	22.279	3.408%
	MAE	12.194	11.589	4.958%

**Table 7:** Optimum number of freezing layers per heating zone.

Heating zone	2	3	4	5	6	7	8	9	10
Number of freezing layers	2	28	2	2	2	2	2	4	24

**Table 8:** RMSE scores for different models applied to nine target zones.

	**GAN-TL**	**Fine-Tune**	**TL-BiLSTM**	**Self-TL-TCN**	**TCN**	**LSTM**	**BiLSTM**	**GRU**	**CNN-LSTM**	**Improvement**
Zone 2	16.376	16.622	26.737	29.923	45.937	46.683	50.034	49.908	57.514	45.27%
Zone 3	7.803	9.137	10.058	12.504	14.817	15.410	14.855	14.968	16.321	37.60%
Zone 4	1.644	1.567	1.744	3.049	3.759	4.365	5.244	4.275	5.074	48.61%
Zone 5	6.200	6.214	8.688	10.048	13.310	11.598	14.776	17.558	14.085	38.30%
Zone 6	3.062	3.204	3.793	5.064	7.017	7.935	7.838	7.804	7.534	39.53%
Zone 7	2.272	2.143	3.211	2.987	6.751	9.271	10.486	9.756	11.129	28.26%
Zone 8	3.560	4.072	5.427	7.096	10.944	12.563	11.771	11.104	11.929	49.83%
Zone 10	3.309	3.037	5.790	7.125	9.850	9.948	9.861	10.113	12.734	57.38%
	**GAN-TL**	**Fine-Tune**	**TL-BiLSTM**	**Self-TL-GRU**	**TCN**	**LSTM**	**BiLSTM**	**GRU**	**CNN-LSTM**	**Improvement**
Zone 9	2.866	2.124	4.063	3.791	6.360	7.106	6.727	6.206	7.064	43.97%

**Table 9:** MAE scores for different models applied to nine target zones.

	**GAN-TL**	**Fine-Tune**	**TL-BiLSTM**	**Self-TL-TCN**	**TCN**	**LSTM**	**BiLSTM**	**GRU**	**CNN-LSTM**	**Improvement**
Zone 2	12.006	12.595	21.536	24.407	38.162	40.790	43.751	42.851	47.471	50.81%
Zone 3	6.116	6.936	8.163	9.092	10.964	12.215	11.420	11.567	12.712	32.73%
Zone 4	1.318	1.295	1.433	2.334	2.996	3.400	4.366	3.440	3.971	44.52%
Zone 5	4.683	4.792	6.988	7.848	10.219	9.640	11.834	14.846	11.427	40.33%
Zone 6	2.418	2.463	2.955	4.080	5.642	6.280	6.173	6.135	6.011	40.73%
Zone 7	1.696	1.680	2.731	2.400	5.354	7.438	8.924	8.573	9.201	30.00%
Zone 8	2.862	3.361	4.979	5.985	9.150	10.171	9.521	9.306	10.091	52.18%
Zone 10	2.448	2.285	3.935	4.724	5.456	6.194	6.261	6.541	7.020	51.63%
	**GAN-TL**	**Fine-Tune**	**TL-BiLSTM**	**Self-TL-GRU**	**TCN**	**LSTM**	**BiLSTM**	**GRU**	**CNN-LSTM**	**Improvement**
Zone 9	2.157	1.631	3.092	3.301	5.298	6.009	5.729	5.071	5.889	50.59%

**Table 10:** The similarity between the generated features and the source domain, as well as the similarity between the original features and the source domain.

Zone	Zone 2	Zone 3	Zone 4	Zone 5	Zone 6	Zone 7	Zone 8	Zone 9	Zone 10
Original	−0.19	−0.15	0.84	0.72	0.58	0.82	0.18	0.72	0.72
Generated	0.90	0.37	0.71	0.88	0.82	0.74	0.80	0.60	0.60

**Table 11:** The complexity of the transfer learning framework and other models.

	Zone 2	Zone 3	Zone 4	Zone 5	Zone 6	Zone 7	Zone 8	Zone 9	Zone 10
TL-TCN	119,937	57,921	119,937	119,937	119,937	119,937	119,937	111,681	62,081
Self-TCN	181,890	181,890	247,938	132,244	181,890	243,906	223,234	173,634	247,938
TCN	123,969	123,969	123,969	123,969	123,969	123,969	123,969	123,969	123,969
LSTM	326,913	97,921	97,921	326,913	97,921	326,913	97,921	97,921	97,921
GRU	245,249	73,473	245,249	245,249	73,473	245,249	245,249	73,473	245,249
BiLSTM	653,825	195,841	195,841	653,825	195,841	653,825	195,841	195,841	195,841
CNN-LSTM	549,221	155,649	155,649	275,877	155,649	275,877	155,649	155,649	231,141

**Table 12:** Running time of each model (the unit is seconds).

	Zone 2	Zone 3	Zone 4	Zone 5	Zone 6	Zone 7	Zone 8	Zone 9	Zone 10
TL-TCN	51	28	50	51	51	52	51	45	31
Self-TCN	125	125	145	101	124	142	138	119	143
TCN	90	91	90	92	90	90	91	92	91
LSTM	703	366	365	700	366	703	365	365	366
GRU	504	360	500	502	358	502	503	348	500
BiLSTM	1413	733	735	1415	733	1413	730	732	732
CNN-LSTM	302	255	256	283	255	285	254	255	280

**Table 13:** Air quality prediction results with daily sampling interval.

	TL-TCN	TL-BiLSTM	Self-TL-TCN	TCN	LSTM	BiLSTM	GRU	CNN-LSTM
RMSE	92.8767	93.6835	93.9478	94.7354	96.4152	95.8696	96.2931	105.0256
MAE	65.3171	65.4864	65.4517	66.2132	66.5143	66.8429	67.4102	75.2448

**Table 14:** RMSE score when zone 3 is the source domain.

	**TL-TCN**	**TL-BiLSTM**	**Self-TL-TCN**	**TCN**	**LSTM**	**BiLSTM**	**GRU**	**CNN-LSTM**	**Improvement**
Zone 2	16.244	26.737	29.923	45.937	46.683	50.034	49.908	57.514	45.71%
Zone 4	2.609	1.744	3.049	3.759	4.365	5.244	4.275	5.074	14.43%
Zone 5	8.528	8.688	10.048	13.310	11.598	14.776	17.558	14.085	15.13%
Zone 6	3.363	3.793	5.064	7.017	7.935	7.838	7.804	7.534	33.59%
Zone 7	2.984	3.211	2.987	6.751	9.271	10.486	9.756	11.129	0.1%
Zone 8	4.767	5.427	7.096	10.944	12.563	11.771	11.104	11.929	32.82%
Zone 10	3.574	5.790	7.125	9.850	9.948	9.861	10.113	12.734	49.84%
	**TL-TCN**	**TL-BiLSTM**	**Self-TL-GRU**	**TCN**	**LSTM**	**BiLSTM**	**GRU**	**CNN-LSTM**	**Improvement**
Zone 9	3.924	4.063	3.791	6.360	7.106	6.727	6.206	7.064	−3.51%

**Table 15:** MAE score when zone 3 is the source domain.

	**TL-TCN**	**TL-BiLSTM**	**Self-TL-TCN**	**TCN**	**LSTM**	**BiLSTM**	**GRU**	**CNN-LSTM**	**Improvement**
Zone 2	14.266	21.536	24.407	38.162	40.790	43.751	42.851	47.471	41.55%
Zone 4	2.179	1.433	2.334	2.996	3.400	4.366	3.440	3.971	6.64%
Zone 5	6.882	6.988	7.848	10.219	9.640	11.834	14.846	11.427	12.31%
Zone 6	2.853	2.955	4.080	5.642	6.280	6.173	6.135	6.011	30.07%
Zone 7	2.234	2.731	2.400	5.354	7.438	8.924	8.573	9.201	18.20%
Zone 8	3.904	4.979	5.985	9.150	10.171	9.521	9.306	10.091	34.77%
Zone 10	2.899	3.935	4.724	5.456	6.194	6.261	6.541	7.020	38.63%
	**TL-TCN**	**TL-BiLSTM**	**Self-TL-GRU**	**TCN**	**LSTM**	**BiLSTM**	**GRU**	**CNN-LSTM**	**Improvement**
Zone 9	3.477	3.092	3.301	5.298	6.009	5.729	5.071	5.889	−5.33%

**Table 16:** Comparison of transfer results of different source domains.

		Zone 2	Zone 4	Zone 5	Zone 6	Zone 7	Zone 8	Zone 9	Zone 10
RMSE	Zone 1	16.622	1.567	6.214	3.204	2.143	4.072	2.124	3.037
	Zone 3	16.244	2.609	8.528	3.363	2.984	4.767	3.924	3.574
MAE	Zone 1	12.595	1.295	4.792	2.463	1.680	3.361	1.631	2.285
	Zone 3	14.266	2.179	6.882	2.853	2.234	3.904	3.477	2.899

**Table 17:** The prediction results of each model in heating furnace 2.

Furnace 2		TL-TCN	Self-TL-TCN	TCN	LSTM	BiLSTM	GRU	CNN-LSTM
Zone 1	RMSE	4.173	6.912	9.188	9.177	10.197	9.227	12.208
	MAE	3.209	5.677	7.718	7.454	8.147	7.303	10.287
Zone 5	RMSE	3.668	4.009	8.143	9.205	9.923	8.199	12.070
	MAE	2.837	3.300	6.739	8.309	8.822	7.307	10.728
Zone 10	RMSE	4.168	9.697	9.725	10.070	10.188	9.879	11.350
	MAE	3.407	8.681	7.889	8.946	8.651	8.976	10.200

**Table 18:** Knowledge transfer between the same equipment at different times.

Furnace 1		TL-TCN	Self-TL-TCN	TCN	LSTM	BiLSTM	GRU	CNN-LSTM
Zone 1	RMSE	5.499	16.417	17.243	21.082	17.368	14.591	20.085
	MAE	4.214	11.442	12.874	18.903	14.805	13.368	17.703
Zone 5	RMSE	5.097	6.196	8.344	9.191	13.456	9.159	9.353
	MAE	4.196	5.327	6.945	7.251	10.175	7.916	6.784
Zone 10	RMSE	2.084	2.264	3.306	3.565	3.458	4.093	4.644
	MAE	1.793	1.903	2.479	2.899	2.855	3.432	3.586

**Table 19:** Knowledge transfer between different equipment at different times.

Furnace 2		TL-TCN	Self-TL-TCN	TCN	LSTM	BiLSTM	GRU	CNN-LSTM
Zone 1	RMSE	7.138	9.898	11.162	10.835	11.418	9.593	11.608
	MAE	5.242	8.268	8.979	9.485	8.947	8.404	9.496
Zone 5	RMSE	11.632	14.522	14.694	14.714	17.596	12.288	15.439
	MAE	8.721	10.711	11.228	10.284	12.971	10.298	11.281
Zone 10	RMSE	4.716	5.446	5.598	7.858	5.681	6.239	8.225
	MAE	3.748	4.245	4.520	6.582	4.626	5.242	6.321

## Appendix A

The appendix mainly supplements the data of heating furnace and the temperature control range required for different steels in actual production. Figure A1 shows the temperature distribution of the target variables for each heating zone.

The heating furnace that we studied this time is a three-stage heating furnace, which is divided into a preheating section, heating section, and soaking section. There are 10 heating zones in the three heating sections. The three heating sections have different temperature requirements. Moreover, the type of billet and the temperature of slab entering the furnace will also lead to different temperatures. The specific requirements for the required temperature are shown in Table A1 and Table A2. However, due to the confidentiality of the company, we did not show the specific steel brand.

**Table A1:** Requirements for temperature control of cold slab furnace.

Representative Brand	Soaking Section (°C)	Heating Section (°C)	Preheating Section (°C)
	09, 10	07, 08 05, 06	04, 03 01, 02
Q ***	1300~1190	1310~1180	1250~1000
S ***	1310~1180	1300~1170	1250~1000
Q ***	1300~1180	1310~1170	1240~1000
X ***	1300~1180	1310~1170	1240~1000

Note: *** is composed of number, quality grade symbol and deoxidation method symbol.

**Table A2:** Requirements for temperature control of hot slab furnace.

Representative Brand	Soaking Section(°C)	Heating Section (°C)	Preheating Section (°C)
	09, 10	07, 08 05, 06	04, 03 01, 02
Q ***	1290~1190	1300~1180	1250~1000
S ***	1300~1180	1290~1170	1250~1000
Q ***	1290~1180	1300~1170	1240~1000
X ***	1290~1180	1300~1170	1240~1000

Note: (A) The slab temperature in the furnace <400 °C is the cold billet, and the slab temperature in the furnace ≥400 °C is the hot billet. (B) The temperature of the furnace in the table is allowed to fluctuate within 30 °C of the instantaneous (<5 min) overrun. *** is composed of number, quality grade symbol and deoxidation method symbol.
